# Factors contributing to emotional distress when caring for children with imperforate anus: a multisite cross-sectional study in China

**DOI:** 10.3389/fmed.2023.1088672

**Published:** 2023-12-07

**Authors:** Meng Lv, Ya Feng-Fang, Yi Wang, Hong Zhen-Xu

**Affiliations:** Children's Hospital, Zhejiang University School of Medicine, Hangzhou, Zhejiang, China

**Keywords:** anus, imperforate, caregiver, distress, cross-sectional studies

## Abstract

**Background:**

Imperforate anus (IA) has a life-long impact on patients and their families. The caregivers of children with IA (CoCIA) might experience distress, which could be detrimental to them physically and mentally. However, there are limitations in the related studies. This study aimed to investigate the prevalence of IA and the associated factors contributing to the distress experienced by CoCIA.

**Methods:**

A cross-sectional study was conducted in three tertiary children's hospitals from November 2018 to February 2019. Distress was assessed using the Chinese version of the Kessler Psychological Distress Scale, and possible determinants were assessed by the Caregiver Reaction Assessment, the Parent Stigma Scale, the Parent Perception of Uncertainty Scale, and the Social Support Scale. Demographic and clinical information was also collected. Multiple regression analysis was performed to explore the association between variables.

**Results:**

Out of 229 CoCIA, 52.9% reported experiencing a high level of distress or above. The data analysis revealed that health problems associated with caregiving, stigma, uncertainty, social support, and children who underwent anal reconstruction surgery 1 year before or earlier could significantly predicate caregivers' distress, and these factors could explain 50.1% of the variance.

**Conclusions:**

The majority of the caregivers of children with IA experience high levels of distress, particularly when their children undergo anal reconstruction surgery 1 year before or earlier. Additionally, health problems related to caregiving, stigma, uncertainty, and low social support could significantly predicate caregivers' distress. It is important for clinical staff to be aware of the prevalent situation of caregivers' distress and to make targeted interventions focused on addressing modifiable factors that should be carried out in family-based care.

## Background

Imperforate anus (IA) is one of the most common types of anorectal malformation (1), with an incidence ranging from 1/2,000 to1/5,000 (2), and its prevalence appears higher in Eastern countries, possibly due to variations in ethnic and medical settings (3). IA is generally categorized into three types: low, intermediate, and high, according to the Wingspread classification (4). Anorectal reconstruction is necessary for all affected children, following which anal dilations are performed until the desired size for their age is achieved (5). Additionally, intermediate- and high-type IA patients require a temporary colostomy and typically undergo surgeries in three stages (6, 7). It is essential for children with IA and their families to have frequent follow-up sessions (8).

Caregivers, who are mostly family members, often experience significant physical, financial, and psychological pressure while providing care (9). Additionally, caregiving is a time-consuming task that may disrupt the daily schedule of caregivers (10). Thus, the abovementioned factors may promote a certain level of negative feelings, such as distress, in caregivers of children with IA (CoCIA) (11), and the presence of negative emotions can have a detrimental effect on the quality of care provided. Therefore, it is important to identify the factors associated with distress to help them more effectively.

It is common to observe stigma in children who are affected (12). However, the family members, especially those responsible for caregiving, may experience stigma due to their close relationship with the patients (13, 14). Stigma usually triggers feelings of shame about their situation, leading to the social isolation of caregivers (15). Furthermore, it adversely affects their mental health, thereby increasing their distress levels (16).

Despite the advancements in surgical procedures over the past several decades, postoperative constipation and fecal and urinary incontinence are highly prevalent among affected individuals (17), and it was reported that CoCIA often feel inadequately informed about the children's prognosis and how to properly manage their bowel functions (1). The aforementioned factors could significantly increase caregivers' perception of uncertainty surrounding the disease (18), thus contributing to higher levels of distress of caregivers (19).

However, studies show that social support is an important protective factor of an individual's psychological health and may have the potential to alleviate caregivers' distress (20). Support from friends, family, or social organizations could alleviate negative emotional reactions experienced by caregivers during IA caregiving (21). Such support might also reduce caregivers' distress. Nevertheless, certain studies have shown that caregivers of children with congenital diseases often lack adequate support (22), implying that CoCIA may also potentially experience lower social support than average.

To date, numerous studies have explored the mental health of children with IA, but there is limited research on the mental wellbeing of families caring for children with IA, which include CoCIA (10). A previous study indicated that approximately 50% of CoCIA reported disruptions in their social lives and family function (1). However, studies related to caregivers' psychological health and its associated factors are currently limited. Therefore, in this study, we aimed to explore the caregivers' distress level and its contributing factors to provide evidence to improve family-based care.

## Methods

### Study design, setting, and sample

This study utilized a cross-sectional design. Sequential participant recruitment was conducted, enrolling caregivers of children receiving treatment for IA based on their arrival order at the hospital between November 2018 and February 2019.

The sample size was calculated using PASS 13.0 software (NCSS, Kaysville, USA). Considering the number of variables and pre-test analysis, the effect size was estimated to be moderate at 0.20, according to the result of the pilot study, and a sample size of 197 caregivers was estimated to achieve the power of 0.8 when α was set as 0.05. Considering a dropout rate of approximately 20% according to our experience, a minimum sample size of 246 caregivers was required for this study.

### Participants

Children who were enrolled in the study were selected if they met the following inclusion criteria: (1) congenital absence of anus or congenital anal fistula, (2) a history of surgical treatment, and (3) those whose guardians had agreed to provide their children's data. Children excluded from the study were as follows:(1) those diagnosed with other life-threatening diseases or severe comorbidity, such as congenital cardiac malformation, and (2) those whose guardians had refused treatment. To participate in this study, caregivers needed to be (1) aged ≥ 18 years (2) have cared for the children for at least 4 weeks, and (3) the primary caregivers, who spent the longest time in caregiving, without any remuneration. The exclusion criteria included candidates (1) who underwent severe life events such as cancer in the past 3 months (a short questionnaire was used for screening) and (2) who refused to take part in this study.

### Measures

#### Sample characteristics

This study examined the characteristics of both caregivers and children. The characteristics of caregivers included their age, gender, marriage, education, work status, income, place of residence, religion, household structure, and relationship with the affected children. On the other hand, the characteristics of children included their age, gender, birth order, the period following anal reconstruction surgery, IA type, and medical insurance. Some general questions were also asked: do you deliberately conceal the children's disease in your social life (yes/no)?; Are you afraid to give birth to another child because the current one was born with an IA (yes/no)?; How frequently do you communicate with medical staff (never/seldom/general/often/always)?; What is the degree of your understanding of the disease (not all/little/some/a lot/very well)?

#### Dependent variable

In our study, distress levels were assessed using the Chinese version of the Kessler Psychological Distress Scale (K10) (23). The K10 is a screening scale with 10 questions that were constructed based on the item response theory models. It was originally designed for use in the annual US National Health Interview Survey (NHIS) to measure non-specific psychological distress experienced by individuals with different mental disorders. The K10 has demonstrated excellent precision within its intended scale distribution range and consistent levels of severity across various sociodemographic subgroups (24). It can be easily administered by participants themselves or through an interviewer, taking only approximately 3 min to complete, and is available for free on a website (www.crufad.org) (25). The K10 is a validated and effective measure of nonspecific psychological distress and is widely utilized in international epidemiological trend surveys (26, 27). Within the K10, respondents were asked to indicate the frequency at which they had experienced mental health-related conditions such as psychological anxiety and stress over the past 30 days. Each item was rated on a scale of 1 to 5, with higher scores indicating greater severity. The total scores ranged from 10 to 50, with higher scores indicating higher levels of distress (28). According to the Victorian Population Health Survey, a score of 10–15 represented a low level of distress while a score of 16–21 indicated a moderate level of distress. A score of 22–29 represented a high level of distress, and a score of 30–50 indicated a very high level of distress (29). We adopted this classification in our study, and the total score of K10 was used for the analysis. The Cronbach's alpha value of K10 was 0.93 in this study.

#### Independent variable

The burden of caregivers was assessed using the Chinese version of the Caregiver Reaction Assessment (CRA) (30). The 24 items on the questionnaire were divided into five dimensions, including impact on health, financial situation, a lack of family support, disrupted daily schedule, and caregivers' esteem. Each dimension was analyzed as a separate factor. The former four dimensions reflected the caregivers' burden, while the last one assessed the positive reaction of caregivers. In this study, the total score of each dimension was used as independent variables (31), and the Cronbach's alpha values were 0.651, 0.802, 0.710, 0.753, and 0.694 for each dimension.

Stigma was measured using the Chinese version of the Parent Stigma Scale (32), which consisted of five items. A 5-point Likert scale was used to assess each item ranging from strongly disagree (1) to strongly agree (5) (33). The total score was used for analysis. In this study, the Cronbach's alpha value was 0.883.

The perception of uncertainty was assessed using the revised Chinese version of the Parent Perception of Uncertainty in Illness Scale (PPUS) (34). The 29 items were ranked from 1 (strongly disagree) to 5 (strongly agree) (34). The total score was calculated as an independent variable. The Cronbach's alpha value was 0.914 in this study.

The Social Support Scale developed by Xiao (35) was used in this study. There were 10 items in total, and higher scores indicated greater level of social support. This classic scale was developed in the Chinese context and is widely used in Chinese studies (36). In this study, Cronbach's alpha value was 0.819, and the total score was summed as an independent factor.

### Data analysis

For qualitative data, the frequency and percentage were used to represent the results. For quantitative data, normally distributed data were described using the mean plus or minus the standard deviation, while non-normally distributed data were described using the median and quartiles. A univariate analysis was conducted with K10 serving as the dependent variable and sample characteristics as the independent variable. If the independent variable satisfies the assumption of normality and homogeneity of variance, the Student's *t*-test was used to compare the two groups, while ANOVA was used to compare the means among multiple groups. However, if the data deviate from a normal distribution or display heteroscedasticity, non-parametric tests such as the Mann-Whitney *U*-test were employed. In the case of non-parametric comparisons involving multiple groups, the Kruskal-Wallis test was utilized. The Spearman's correlation coefficients were calculated to assess the potential correlations between distress and variables such as CRA, stigma, PPUS, and social support. Multiple linear regression was performed to analyze the associated factors of caregivers' distress. Statistical significance was assessed at the 5% level (*p* < 0.05).

### Procedure

Ethical approval was obtained for this study (2018-IRB-081). A pilot test of 30 primary caregivers was conducted to ensure that the questionnaire contents could be properly understood. In our formal study, we utilized an online survey to collect data. Specifically, the principal investigator set up three separate WeChat groups, one for each center involved in our multi-center study. In each group, a pediatric surgeon and a wound ostomy continence nurse were invited to act as consultants. To ensure maximum participation, we first added each caregiver participant on WeChat. If they expressed a willingness to complete the survey, the principal investigator would contact them online and provide them with the link to the online survey. This link was composed of two parts: an electronic consent form and a set of questionnaires. Caregivers could only proceed to the questionnaires after clicking on “I have read all the content and agree to participate in the study”. Participants were able to complete the survey either in a separate room at the clinic of pediatric surgery or in the comfort of their own homes, depending on their preference and convenience. For illiterate candidates, researchers read and explained every item to ensure inclusivity. All caregivers were informed about the WeChat groups and given a brief introduction to the study. They were also offered the option to join the groups, regardless of whether they would like to participate in the survey. These groups provided a forum for patients and caregivers to connect with others who had comparable experiences. They were able to discuss any questions that came up while filling out the questionnaires and receive additional information and support. It is also noteworthy that these WeChat groups continued to operate after the project had concluded, providing ongoing medical advice and social support to caregivers.

## Results

### Sample characteristics

The data analysis was conducted using 229 completed questionnaires from primary caregivers (response rate = 90.16%). Out of the initial pool of 254 participants who were eligible for this study, 18 individuals declined to participate citing the reason as the lack of interest or unwillingness to discuss their child's disease. Additionally, seven participants were excluded from the study due to incomplete questionnaires. Based on the sample size of 229, an effective size of 0.20, and an α-value set at 0.05, the actual statistical power of this study was calculated to be 0.857. The characteristics of both the caregivers and children are presented in Table 1. The age of the caregivers who participated in the study ranged from 18 to 67 years, with a median age of 30 (interquartile range: 28–36). The age of children who participated in the study ranged from 0.08 to 6.37 years old, with a median age of 0.87 years (interquartile range: 0.37–1.78).

**Table 1:** Descriptive statistics and univariate analysis (*N* = 229).

**Item**	***N* (%)**	**K10**	***χ^2^*/*t*/z**	***P***
**Caregiver**				
Age			0.402	0.670
< 30	100 (43.7)	22.80 ± 7.33		
30–40	106 (46.3)	23.47 ± 7.30		
>40	23 (10.0)	22.13 ± 8.56		
Gender			0.677	0.499
Men	41 (17.9)	23.76 ± 6.70		
Women	188 (82.1)	22.89 ± 7.58		
Marriage			−0.087	0.930
Married	222 (96.9)	23.04 ± 7.49		
Other	7 (3.1)	23.29 ± 5.44		
Education			0.543	0.653
Primary school or below	17 (7.4)	24.06 ± 6.83		
Junior high school	68 (29.7)	23.77 ± 6.81		
High school	45 (19.7)	22.91 ± 6.81		
University/college or above	99 (43.2)	23.04 ± 7.42		
Occupation			1.725	0.181
Part-time job	32 (14.0)	25.22 ± 6.50		
Full-time job	84 (36.7)	22.39 ± 7.72		
Unemployed	113 (49.3)	22.91 ± 7.40		
Relationship with children			1.636	0.197
Mother	178 (77.7)	22.76 ± 7.44		
Father	44 (19.2)	24.64 ± 7.00		
Others	7 (3.1)	20.29 ± 8.90		
Income (RMB)				
< 8,000	111 (48.5)	24.56 ± 6.75	−3.465	0.001
≥8,000	118 (51.5)	20 (16, 25)		
Inhabitant			1.692	0.187
City	61 (26.6)	21.72 ± 7.61		
Suburban	76 (33.2)	22.99 ± 7.16		
Countryside	92 (40.2)	23.97 ± 7.46		
Household structure			−1.097	0.274
Extended family	150 (65.5)	22.65 ± 6.72		
Nuclear family	79 (34.5)	23.79 ± 8.60		
Religion			1.250	0.212
Yes	71 (31.0)	23.96 ± 8.62		
No	158 (69.0)	22.63 ± 6.81		
Medical insurance			−1.633	0.104
Yes	173 (75.5)	22.59 ± 7.29		
No	56 (24.5)	24.45 ± 7.71		
Concealment behavior			2.522	0.012
Yes	161 (70.3)	23.84 ± 7.49		
No	68 (29.7)	21.16 ± 6.96		
Afraid to have another child			3.273	0.001
Yes	139 (60.7)	24.31 ± 7.59		
No	90 (39.3)	21.09 ± 6.75		
Communication frequency with medical staff			1.569	0.118
General or below	138 (60.3)	23.67 ± 7.24		
Often or above	91 (39.7)	22.10 ± 7.64		
Understanding of IA			0.154	0.878
Some or below	176 (76.9)	23.09 ± 7.40		
A lot or above	53 (23.1)	22.91 ± 7.57		
**Children**				
Age (y)			−0.849	0.397
< 2	189 (82.5)	22.85 ± 7.39		
≥2	40 (17.5)	23.95 ± 7.62		
Gender			0.062	0.951
Men	154 (67.2)	23.07 ± 7.18		
Women	75 (32.8)	23.00 ± 7.96		
Birth order			−0.849	0.397
1	102 (44.5)	22.58 ± 7.22		
≥2	127 (55.5)	23.42 ± 7.60		
Time since anal reconstruction surgery (y)			−1.995	0.047
< 1	128 (55.9)	22.18 ± 7.37		
≥1	101 (44.1)	24.14 ± 7.39		
IA type			5.136	0.007
Low	124 (54.1)	21.64 ± 6.63		
Intermediate	41 (17.9)	24.29 ± 9.60		
High	64 (27.9)	24.97 ± 6.82		
Combined with other abnormalities			1.687	0.093
Yes	94 (41.0)	24.03 ± 7.79		
No	135 (59.0)	22.36 ± 7.11		

### Measurement results

The mean distress score of caregivers was 23.04. Table 2 presented the distribution of distress levels, which were as follows: low-−34 (14.8%), moderate−74 (32.3%), high−75 (32.8%), and very high−46 (20.1%). The total score and average score of the items for the measurement scales, including K10, CRA, PPUS, social support, and stigma, were shown in Table 3.

**Table 2:** Category of K10 score (*N* = 229).

**Degree of distress**	***N* (%)**
Low	34 (14.8)
Moderate	74 (32.3)
High	75 (32.8)
Very high	46 (20.1)

**Table 3:** Descriptive statistics for measurement scales (*N* = 229).

**Item**	**Total score (range)**	**Item average (range)**
K10	23.04 ± 7.42 (10.00–50.00)	2.30 ± 0.74 (1.00–5.00)
CRA		
Health problem	10.74 ± 2.63 (4.00–18.00)	2.69 ± 0.66 (1.00–4.50)
Financial problem	8.77 ± 2.77 (3.00–15.00)	2.92 ± 0.92 (1.00–5.00)
Lack of family support	11.22 ± 3.19 (5.00–22.00)	2.24 ± 0.64 (1.00–4.40)
Disrupted schedule	18.00 ± 3.60 (5.00–25.00)	3.60 ± 0.71 (1.00–5.00)
Self-esteem	29.01 ± 3.20 (20.00–35.00)	4.14 ± 0.46 (2.86–5.00)
Stigma	14.98 ± 4.81(5.00–25.00)	3.00 ± 0.96 (1.00–5.00)
PPUS	72.58 ± 14.06 (29.00–102.00)	2.59 ± 0.50 (1.04–3.64)
Social support	40.33 ± 8.36 (16.00–62.00)	2.88 ± 0.60 (1.14–4.43)

K10, Kessler Psychological Distress Scale; CRA, Caregiver Reaction Assessment; PPUS, Parent Perception of Uncertainty Scale.

### Univariate analysis

The univariate analysis identified the significant differences in income, the period following anal reconstruction surgery, IA type, concealment behavior, and fear of having another child (Table 1). The relationships between CRA, stigma, PPUS, social support, and distress are shown in Table 4. The correlation analysis showed that there were statistically significant correlations between CRA, stigma, PPUS, and social support with K10.

**Table 4:** Correlations (*r*) between CRA, stigma, PPUS, social support, and K10.

	**K10**	**CRA health problem**	**CRA financial problem**	**CRA lack of family support**	**CRA disrupted schedule**	**CRA self-esteem**	**PPUS**	**Social support**	**Stigma**
K10	1.00	0.623^**^	0.435^**^	0.285^**^	0.322^**^	−0.250^**^	0.529^**^	−0.424^**^	0.397^**^
CRA								
Health problem	0.623^**^	1.00	0.506^**^	0.277^**^	0.424^**^	−0.270^**^	0.407^**^	−0.343^**^	0.314^**^
Financial problem	0.435^**^	0.506^**^	1.00	0.330^**^	0.443^**^	−0.217^**^	0.489^**^	−0.378^**^	0.233^**^
Lack of family support	0.285^**^	0.277^**^	0.330^**^	1.00	0.263^**^	−0.251^**^	0.358^**^	−0.438^**^	0.163^*^
Disrupted schedule	0.322^**^	0.424^**^	0.443^**^	0.263^**^	1.00	00.099	0.201^**^	−0.275^**^	0.283^**^
Self-esteem	-0.250^**^	−0.270^**^	−0.217^**^	−0.251^**^	00.099	1.00	−0.332^**^	0.305^**^	−0.185^**^
PPUS	0.529^**^	0.407^**^	0.489^**^	0.358^**^	0.201^**^	−0.332^**^	1.00	−0.364^**^	0.283^**^
Social support	-0.424^**^	−0.343^**^	−0.378^**^	−0.438^**^	−0.275^**^	0.305^**^	−0.364^**^	1.00	−0.293^**^
Stigma	0.397^**^	0.314^**^	0.233^**^	0.163^*^	0.283^**^	−0.185^**^	0.283^**^	−0.293^**^	1.00

CRA, Caregiver Reaction Assessment; PPUS, Parent Perception of Uncertainty Scale.

** Correlation is significant at the 0.01 level (2-tailed).

* Correlation is significant at the 0.05 level (2-tailed).

### Multiple regression analysis

The associated factors of caregivers' distress were explored using the multiple regression analysis. Table 5 displays the results of the multiple regression analysis of significant univariable factors. Table 6 reveals that independent factors contributing to the distress of IA caregivers included health problems pertaining to caregiving (*B* = 1.186), PPUS (*B* = 0.132), stigma (*B* = 0.243), social support (*B* = −0.104), and children who underwent anal reconstruction surgery ≥1 year (*B* = 1.445). These factors accounted for a variance of 50.1% of caregivers' distress.

**Table 5:** Multiple regression analysis of significant univariable factors (*N* = 229).

**Item**	***B***	***SE***	***SB***	***t***	***P***	***VIF***
Constant	−0.553	6.204		−0.089	0.929	
Stigma	0.228	0.083	0.148	2.745	0.007	1.311
CRA						
Health problem	1.333	0.183	0.472	7.278	< 0.001	1.898
Finance problem	−0.125	0.175	−0.047	−0.716	0.475	1.912
Lack of family support	−0.011	0.134	−0.005	−0.082	0.935	1.501
Disrupted schedule	−0.047	0.131	−0.022	−0.356	0.722	1.761
Self-esteem	0.119	0.127	0.051	0.934	0.351	1.354
Social support	−0.104	0.053	−0.117	−1.966	0.051	1.598
PPUS	0.128	0.032	0.242	3.978	< 0.001	1.675
Time since anal reconstruction surgery	1.385	0.724	0.093	1.914	0.057	1.062
Income	−1.249	0.804	−0.084	−1.553	0.122	1.327
Concealment behavior	−0.944	0.832	−0.058	−1.135	0.258	1.188
Afraid to have another child	0.167	0.776	0.011	0.216	0.829	1.18
IA type	0.15	0.436	0.018	0.344	0.731	1.178

R^2^ = 0.524, R${\;}_{\text{ad}}^{2}$ = 0.495, F = 18.178, P < 0.01. Durbin-Watson = 2.020.

**Table 6:** Multiple regression analysis of independent influencing factors (*N* = 229).

**Item**	***B***	***SE***	**S*B***	***t***	***P***	***VIF***
Constant	−0.809	3.711	–	−0.218	0.828	–
CRA Health problem	1.186	0.153	0.420	7.747	< 0.01	1.341
PPUS	0.132	0.028	0.251	4.652	< 0.01	1.327
Stigma	0.243	0.078	0.157	3.105	0.002	1.171
Social support	−0.104	0.047	−0.118	−2.212	0.028	1.289
Time since surgery	1.445	0.704	0.097	2.051	0.041	1.017

R^2^ = 0.512, R${\;}_{\text{ad}}^{2}$ = 0.501, F = 46.714, P < 0.01. Durbin-Watson = 2.011.

## Discussion

This study investigated the extent of distress experienced by CoCIA and identified its contributing factors. On average, the caregivers obtained a K10 score of 23.04, which indicates a high level of distress. This score was much higher than both the average population score (15.42) (37) and the score for patients with chronic illness (20.25) (38). According to a study, the K10′s cutoff point was found to be 16, which falls under the moderate classification category. The study also found that, when higher scores were observed on the scale, the level of psychological distress reported was proportionally higher as well (39). In this study, it was found that approximately 85.15% of the caregivers scored at least 16 points, which suggested that professional psychological consultation should be recommended for this population. Caregivers play a crucial role in establishing a connection between the affected children and medical staff; however, if they experience psychological distress, the quality of care given to IA children may be compromised due to ineffective cooperation with the medical staff (14). Therefore, clinical medical staff should pay more attention to the caregivers' mental health, screen out the high-risk population with effective tools, such as K10, and cooperate with professional psychiatrists.

Different stages of IA diagnosis have an effect on caregivers' psychological state (10). According to our study, children who underwent surgery for IA for 1 year before or earlier were found to be significantly associated with higher levels of caregivers' distress, highlighting their role as a significant predictor, that is, the longer the caregiving duration, the greater the possibility of burnout (40). One possible explanation might be that caregiving for a short period can be manageable, but over a long period of time, it can have a detrimental effect on the psychological health of caregivers and ultimately result in burnout. Therefore, clinical nurses and surgeons should pay particular attention to CoCIA with a history of anal reconstruction surgery 1 year before or earlier. While caregiving tasks eventually reduce at the end of surgical therapy, distress levels may, on the other hand, increase due to the long duration of caregiving.

Health problems can play a significant role in causing distress for IA caregivers. Caring for a child with chronic illness can be a challenging task that imposes a considerable amount of stress on the caregivers and can have a negative effect on the physical health of caregivers (41). In a previous study, it was found that caregivers of children with chronic illness experience more health problems compared to caregivers of healthy children, and this can be solely attributed to the challenges of caregiving (42). Moreover, the health problems could also negatively influence caregivers' mental health and increase their distress level, which is harmful to both the caregivers and their children (43). Thus, medical staff could provide caregivers with more health-keeping knowledge while treating the affected children and encourage caregivers to ask for medical help if necessary. Additionally, it is important to teach nursing skills such as ostomy-care experience and dietary recommendations to enhance caregivers' illness management abilities, which may potentially alleviate caregiving burden and reduce the caregivers' health problems (44).

Perceiving uncertainty of the children's illness improvement is an important determinant of IA caregivers' distress. The persistent uncertainty could lead to higher levels of anxiety and distress among caregivers, thus negatively affecting their overall wellbeing (19, 45). However, some studies indicated that caregivers might also benefit from embracing uncertainty, as acknowledging it opens up the possibility for positive prognoses in the affected children, which could also be viewed as an opportunity for a favorable outcome (46). However, the impact of uncertainty on caregivers' distress usually depends on their individual perspectives and personal viewpoints. On the one hand, as a member of medical staff, we could provide additional information to support caregivers to mitigate their unnecessary anxiety about their children and reduce the negative effect of uncertainty (45). On the other hand, encouraging caregivers to focus on the benefits that can come from uncertainty could help promote optimism and a more positive outlook on the prognosis. Stigma is also a determinant of caregivers' distress. Since the children are usually unaware of their situation, particularly when they are young, the caregivers may experience higher perceived stigma and social discrimination since the burden falls solely on caregivers (16). The stigma causes them to experience more distress because of their personal disapproval and self-depreciation as well as poses a barrier for the caregivers to seek help from medical services, which might result in undermining the quality of the care given (47). Thus, interventions focused on stigma should be implemented. Studies have shown that providing detailed explanations about the disease, sharing testimonies of individuals living with the stigma, and incorporating active-learning exercises contribute to mitigating stigmatization, thus reducing caregivers' distress (47).

According to the results of this study, it was found that social support is a determinant of distress among IA caregivers. Social support plays a significant protective role in individual psychological health, potentially reducing caregivers' distress (20). Medical staff should motivate caregivers to seek additional social support, such as encouraging them to seek help from their family members, and ensuring they have increased access to contact medical staff for further inquiries. Peer support can be harnessed through initiatives such as establishing IA-related communication groups to provide assistance (48).

## Conclusions

CoCIA experience a high level of distress, especially when their children undergo anal reconstruction surgery 1 year before or earlier. This heightened distress is linked to health problems arising from caregiving, increased perception of stigma and uncertainty, and a lower level of social support. Therefore, it is crucial for medical staff to pay attention to the continuing needs of these caregivers during follow-up appointments, even after the patients have completed their surgical therapy. Additionally, some measures aiming to improve the caregivers' health condition and reduce their stigma should be implemented. We should also provide more medical information to alleviate caregivers' anxiety caused by uncertainty, encourage them to be optimistic, and help them find greater social support to promote their psychological health.

## Limitations

This study, as any other study, has its limitations. First, we used an outdated classification system. During the data collection period, the Wingspread classification was still being used as the standard in the Chinese medical system. Unfortunately, due to restricted access to patient data, we were unable to obtain detailed descriptions of the disease to accurately reclassify our patients according to updated systems such as the Krickenbeck classification. Second, due to the correlational nature of the evidence, we could not ascertain the direction of some associations. For example, distress resulting from prolonged caregiving might, in turn, negatively affect the health of caregivers. Therefore, prospective studies are required to identify the direction of associations. Finally, we may have overlooked some important factors that could potentially contribute to caregivers' distress, such as family function, which is regarded as a crucial factor in personal psychological health.

## Data availability statement

The raw data supporting the conclusions of this article will be made available by the authors, without undue reservation.

## Ethics statement

The studies involving humans were approved by Zhejiang University School of Medicine, China. The studies were conducted in accordance with the local legislation and institutional requirements. Written informed consent for participation in this study was provided by the participants' legal guardians/next of kin.

## Author contributions

YF-F conceived and supervised the study and extracted data. ML extracted data, performed data analysis, and developed the manuscript. YW performed data analysis and provided input on manuscript development. HZ-X helped write the manuscript and provided critical input on its revisions. All authors contributed to the article and approved the submitted version.

## References

[1] Cairo S Gasior A Rollins M Rothstein D. Challenges in transition of care for patients with anorectal malformations: a systematic review and recommendations for comprehensive care. Dis Colon Rectum. (2018) 61:390–9. 10.1097/DCR.0000000000001033 29420431

[2] Steeg Hvd Botden S Sloots C Steeg Avd Broens P Heurn Lv Travassos D Rooij Iv Blaauw Id: Outcome in anorectal malformation type rectovesical fistula: a nationwide cohort study in The Netherlands. J Pediat Surg. (2016) 51:1229–33. 10.1016/j.jpedsurg.2016.02.002 26921937

[3] Gangopadhyay AN Pandey V. Controversy of single versus staged management of anorectal malformations. Indian J Pediatr. (2017) 84:636–42. 10.1007/s12098-017-2373-6 28600661

[4] Steeg Hvd Schmiedeke E Bagolan P Broens P Demirogullari B Garcia-Vazquez A. European consensus meeting of ARM-Net members concerning diagnosis and early management of newborns with anorectal malformations. Techniq Coloproctol. (2015) 19:181–5. 10.1007/s10151-015-1267-8 25609592 PMC4369584

[5] Jenetzky E vanRooij IALM Aminoff D Schwarzer N Reutter H Schmiedeke E. The challenges of the European anorectal malformations-net registry. Eur J Pediat Surg. (2015) 25:481–7. 10.1055/s-0035-1569149 26642384

[6] Baayen C Feuillet F Clermidi P Crétolle C Sarnacki S Podevin G. Validation of the French versions of the Hirschsprung's disease and Anorectal malformations Quality of Life (HAQL) questionnaires for adolescents and adults. Health Qual Life Outc. (2017) 15:24. 10.1186/s12955-017-0599-7 28129770 PMC5273813

[7] Nam SH Kim DY Kim SC. Can we expect a favorable outcome after surgical treatment for an anorectal malformation? J Pediatr Surg. (2016) 51:421–4. 10.1016/j.jpedsurg.2015.08.048 26572852

[8] Goudarzi Z Askari M Seyed-Fatemi N Asgari P Mehran A. The effect of educational program on stress, anxiety and depression of the mothers of neonates having colostomy. J Matern Fetal Neonatal Med. (2016) 29:3902–5. 10.3109/14767058.2016.1152242 26864254

[9] Al-Gamal E Long T Shehadeh J. Health satisfaction and family impact of parents of children with cancer: a descriptive cross-sectional study. Scand J Caring Sci. (2019) 33:815–23. 10.1111/scs.12677 30866084

[10] Witvliet MJ Bakx R Zwaveling S. Dijk THv, Steeg AFWvd: Quality of life and anxiety in parents of children with an anorectal malformation or Hirschsprung disease: the first year after diagnosis. Eur J Pediat Surg. (2016) 26:002–6. 10.1055/s-0035-1559885 26382660

[11] Majestic C Eddington KM. The impact of goal adjustment and caregiver burden on psychological distress among caregivers of cancer patients. Psychooncology. (2019) 28:1293–300. 10.1002/pon.5081 30946499

[12] Ojmyr-Joelsson M Nisell M Frenckner B Rydelius P-A Christensson K. A gender perspective on the extent to which mothers and fathers each take responsibility for care of a child with high and intermediate imperforate anus. J Pediatr Nurs. (2009) 24:207–15. 10.1016/j.pedn.2007.09.004 19467434

[13] Hamlington B. E.lvey L, Brenna E, Biesecher LG, B.Biesecker B, Sapp J. Characterization of courtesy stigma perceived by parents of overweight children with bardet-biedl syndrome. PLoS ONE. (2015) 10:e0140705. 10.1371/journal.pone.0140705 26473736 PMC4608820

[14] Chang C Su JA Tsai CS Yen C Liu JH Lin CY. Rasch analysis suggested three unidimensional domains for Affiliate Stigma Scale: additional psychometric evaluation. J Clin Epidemiol. (2015) 68:674–83. 10.1016/j.jclinepi.2015.01.018 25748074

[15] Li J Mo P Wu A Lau J. Roles of self-stigma, social support, and positive and negative affects as determinants of depressive symptoms among HIV infected men who have sex with men in China. AIDS Behav. (2017) 21:261–73. 10.1007/s10461-016-1321-1 26896120 PMC4992470

[16] Zhou T Wang Y Yi C. Affiliate stigma and depression in caregivers of children with Autism Spectrum Disorders in China: Effects of self-esteem, shame and family functioning. Psychiatry Res. (2018) 264:260–5. 10.1016/j.psychres.2018.03.071 29655969

[17] Lane VA Nacion KM Cooper JN Levitt MA Deans KJ Minneci P. Determinants of quality of life in children with colorectal diseases. J Pediatr Surg. (2016) 51:1843–50. 10.1016/j.jpedsurg.2016.08.004 27586859

[18] Giuliani S Decker E Leva E Riccipetitoni G Bagolan P. Long term follow-up and transition of care in anorectal malformations: an international survey. J Pediatr Surg. (2016) 51:1450–7. 10.1016/j.jpedsurg.2016.03.011 27114308

[19] Aldaz BE Hegarty RSM Conner TS Perez D Treharne GJ. Is avoidance of illness uncertainty associated with distress during oncology treatment? A daily diary study. Psychol Health. (2019) 34:422–37. 10.1080/08870446.2018.1532511 30607991

[20] Choi E Yoon S Kim J Park H Kim J Yu E. Depression and distress in caregivers of children with brain tumors undergoing treatment: psychosocial factors as moderators. Psychooncology. (2016) 25:544–50. 10.1002/pon.3962 26426911

[21] Okuyama J Funakoshi S Amae S Kamiyama T Ueno T Hayashi Y. Coping patterns in a mother of a child with multiple congenital anomalies: a case study. J Intens Criti Care. (2017) 3:1–6. 10.21767/2471-8505.100075

[22] Jacobs R Boyd L Brennan K Sinha CK Giuliani S. The importance of social media for patients and families affected by congenital anomalies: a Facebook cross-sectional analysis and user survey. J Pediatr Surg. (2016) 51:1766–71. 10.1016/j.jpedsurg.2016.07.008 27522307

[23] Zhou C Chu J Wang T Peng Q He J Zheng W. Reliability and validity of 10-item Kessler Scale (K10) Chinese version in evaluation of mental health status of Chinese population. Chin J Clin Psychol. (2008) 16:627–9.

[24] Kessler RC Andrews G Colpe LJ Hiripi E Mroczek DK Normand SL. Short screening scales to monitor population prevalences and trends in non-specific psychological distress. Psychol Med. (2002) 32:959–76. 10.1017/S0033291702006074 12214795

[25] Kessler RC Barker PR Colpe LJ Epstein JF Gfroerer JC Hiripi E. Screening for serious mental illness in the general population. Arch Gen Psychiatry. (2003) 60:184–9. 10.1001/archpsyc.60.2.184 12578436

[26] Sunderland M Slade T Stewart G Andrews G. Estimating the prevalence of DSM-IV mental illness in the Australian general population using the Kessler Psychological Distress Scale. Aust N Z J Psychiatry. (2011) 45:880–9. 10.3109/00048674.2011.606785 21875309

[27] Furukawa TA Kawakami N Saitoh M Ono Y Nakane Y Nakamura Y. The performance of the Japanese version of the K6 and K10 in the World Mental Health Survey Japan. Int J Methods Psychiatr Res. (2008) 17:152–8. 10.1002/mpr.257 18763695 PMC6878390

[28] Chan SM Fung TCT. Reliability and validity of K10 and K6 in screening depressive symptoms in Hong Kong adolescents. Vulnerable Child Youth Stud. (2014) 9:75–85. 10.1080/17450128.2013.861620

[29] Victorian Population Health Survey. Victorian Population Health Survey 2008 vol. 26. Melbourne, Victoria: Department of Health. (2008).

[30] Yaping Z Yan L Huiqin W. Validity and reliability research of Chinese edition of caregiver reaction assessment. Chin J Nurs. (2008) 43:856–9. 10.3761/j.issn.0254-1769.2008.09.042

[31] Petrinec A Burant C Douglas S. Caregiver reaction assessment: psychometric properties in caregivers of advanced cancer patients. Psychooncology. (2016) 26:1–3. 10.1002/pon.4159 27237457

[32] Wang D Jia Y Gao W Chen S Li M Hu Y. Relationships between stigma, social support, and distress in caregivers of Chinese children with imperforate anus: a multicenter cross-sectional study. J Pediat Nus. (2019) 49:e15–e20. 10.1016/j.pedn.2019.07.008 31378408

[33] Austin J MacLeod J Dunn D Shen J Perkins S. Measuring stigma in children with epilepsy and their parents: instrument development and testing. Epilep Behav. (2004) 5:472–82. 10.1016/j.yebeh.2004.04.008 15256183

[34] Jiaxuan M Wanhua X Chunhua M Yeqing D Lili D. Initial revision of Chinese version of parents' perception of uncertainty scale. Chin J Pract Nurs. (2013) 29:46–50. 10.3760/cma.j.issn.1672-7088.2013.28.023

[35] Xiao S. The theoretical basis and research application of Social Support Scale. J Clini Psychol Med. (1994) 1994:98–100.

[36] Yang Y Zhang B Meng H Liu D Sun M. Mediating effect of social support on the associations between health literacy, productive aging, and self-rated health among elderly Chinese adults in a newly urbanized community. Medicine. (2019) 98:1–8. 10.1097/MD.0000000000015162 31008936 PMC6494366

[37] Chen J. Some people may need it, but not me, not now: seeking professional help for mental health problems in Urban China. Transcult Psychiat. (2018) 55:754–74. 10.1177/1363461518792741 30113276

[38] Xu M Markström U Lyu J Xu L. Survey on tuberculosis patients in rural areas in China: tracing the role of stigma in psychological distress. Int J Environ Res Public Health. (2017) 14:1–9. 10.17504/protocols.io.i2fcgbn 28976922 PMC5664672

[39] Peltzer K Naidoo P Matseke G Louw J McHunu G Tutshana B. Prevalence of psychological distress and associated factors in tuberculosis patients in public primary care clinics in South Africa. BMC Psychiatry. (2012) 12:1–9. 10.1186/1471-244X-12-89 22839597 PMC3410814

[40] Nguyen JT Roberts C Thorpe CT Thorpe JM Hogan SL McGregor J. Economic and objective burden of caregiving on informal caregivers of patients with systemic vasculitis. Musculoskeletal Care. (2019) 17:282–7. 10.1002/msc.1394 30901158 PMC6591058

[41] Riffin C Ness PHV. L.Wolff J, Fried T. Multifactorial examination of caregiver burden in a national sample of family and unpaid caregivers. J Am Geriatr Soc. (2019) 67:277–83. 10.1111/jgs.15664 30452088 PMC6367031

[42] Brehaut JC Guèvremont A Arim RG Garner RE Miller AR McGrail KM. Changes in caregiver health in the years surrounding the birth of a child with health problems: administrative data from British Columbia. Med Care. (2019) 57:369–76. 10.1097/MLR.0000000000001098 30908379

[43] Venkataramani M Cheng TL Solomon BS Pollack CE. Caregiver health promotion in pediatric primary care settings: results of a national survey. J Pediatr. (2017) 181:1–7.e2. 10.1016/j.jpeds.2016.10.054 27837952

[44] Jingting W Nengliang Y Yuanyuan W Fen Z Yanyan L Zhaohui G. Developing “Care Assistant”: A smartphone application to support caregivers of children with acute lymphoblastic leukaemia. J Telemed Telecare. (2016) 22:163–71. 10.1177/1357633X15594753 26271029

[45] Szulczewski L Mullins LL Bidwell SL Eddington AR Pai ALH. Meta-analysis: caregiver and youth uncertainty in pediatric chronic illness. J Pediatr Psychol. (2017) 42:395–421. 10.1093/jpepsy/jsw097 28177514 PMC6440270

[46] Bell M Biesecker BB Bodurtha J Peay HL. Uncertainty, hope, and coping efficacy among mothers of children with Duchenne/Becker muscular dystrophy. Clini Genet. (2019) 95:677–83. 10.1111/cge.13528 30847900 PMC6529261

[47] Nyblade L Stockton MA Giger K Bond V Ekstrand ML Lean RM. Stigma in health facilities: why it matters and how we can change it. BMC Med. (2019) 17:1–15. 10.1186/s12916-019-1256-2 30764806 PMC6376713

[48] Sari BA Demirogullari B Ozen O iseri E Kale N Basaklar C. Quality of life and anxiety in Turkish patients with anorectal malformation. J Paediatr Child Health. (2014) 50:107–11. 10.1111/jpc.12406 24134432

